# Case Report: Benefits of LSVT LOUD in a multilingual patient with hypokinetic-hyperkinetic dysarthria and suspected progressive supranuclear palsy

**DOI:** 10.3389/fresc.2024.1421730

**Published:** 2024-07-18

**Authors:** Amanda Sebestyen, Allison Hilger, Holly Kleiber

**Affiliations:** Department of Speech, Language, and Hearing Sciences, University of Colorado, Boulder, CO, United States

**Keywords:** dysarthria, Lee Silverman Voice Treatment (LSVT), speech therapies, progressive supranuclear palsy, multilingual

## Abstract

**Purpose:**

This case study measured how well the Lee Silverman Voice Treatment (LSVT) improved vocal features, intelligibility, and communicative effectiveness for a multilingual participant with hypokinetic-hyperkinetic dysarthria secondary to suspected progressive supranuclear palsy. LSVT treatment was chosen for the participant due to the strengths and deficits he presented with prior to treatment, and for the anticipated challenges in treatment that may arise from the presence of multilingualism and impaired cognitive functioning.

**Methods:**

A multilingual patient in their 60's (English, Spanish, and French) with hypokinetic-hyperkinetic dysarthria secondary to suspected progressive supranuclear palsy completed the standard treatment sessions for LSVT. Assessment measures were taken at baseline, immediately post-treatment, and three-months post-treatment.

**Results:**

Improvements were measured in vocal quality, vocal loudness, intelligibility, and communicative effectiveness immediately post-treatment. Three months post-treatment, improvements in vocal quality and intelligibility were maintained.

**Conclusion:**

This case study illustrates that LSVT may be a beneficial treatment for complex clients who are multilingual and present with complex comorbidities and cognitive deficits. LSVT resulted in some meaningful changes in vocal quality, intelligibility, and communicative effectiveness for this individual. Clinicians who work with complex patients may wish to consider the theoretical underpinnings of LSVT, client profile, areas of client need, and ability and desire to complete an intensive treatment program to determine if trialing LSVT is appropriate. The use of LSVT with complex clients may yield positive outcomes.

## Introduction

The Lee Silverman Voice Treatment (LSVT) was originally designed as a speech treatment for patients with hypokinetic dysarthria resulting from Parkinson's Disease (PD) (1). PD often reduces speech intelligibility because of vocal fold bowing (2), decreased range of articulatory motion (3), and a lack of respiratory support (4). The overarching goal of LSVT is to enhance intelligibility by improving laryngeal closure, increasing articulatory range of motion, and improving respiratory support (1, 5). LSVT achieves these goals by utilizing the principles of motor learning and neural plasticity with a simple cue of speaking loudly with a high dosage of treatment. There is increasing evidence that LSVT is effective for populations beyond PD (6, 7) as well as for non-English speakers (8). We conducted a case study on LSVT for a multilingual man with hypokinetic-hyperkinetic dysarthria secondary to suspected progressive supranuclear palsy.

LSVT follows a high dosage and high intensity standardized protocol (9). Clients complete sixteen one-hour treatment sessions delivered across four consecutive days per week for four weeks. Each session consists of four daily exercises that remain the same throughout treatment. In all LSVT treatment activities there is a single focus: “be loud.” This focused, simple cue promotes changes across speech subsystems, and its simplicity is ideal for patients with cognitive deficits. LSVT further minimizes cognitive load during treatment with modeling and shaping techniques. Clients are simply instructed by the clinician to “do what I do” and are provided with cueing for loudness rather than given lengthy explanations.

There is increasing evidence that LSVT is beneficial for non-English speakers. Moya-Galé and colleagues (2018) found that LSVT improved conversational intelligibility in Castilian Spanish speakers with hypokinetic dysarthria secondary to PD. In Whitehill et al. (10), LSVT improved loudness and intonation in Cantonese speakers with idiopathic PD. However, there has been little research on the benefit of LSVT for multilingual speakers, particularly for improvement in production of a second language. Given that English has become a global language and most users of English are non-native speakers (11), it is important to determine the benefit of LSVT for second-language speakers of English. In our case study, we assess improvements in speech production in English for a multilingual man whose native language is French.

Additionally, there is evidence that LSVT is beneficial for conditions beyond PD, such as Down Syndrome (6), multiple sclerosis (12, 13), post-stroke dysarthria (7), and, of relevance to the current study, supranuclear palsy (14). These studies support the hypothesis that LSVT encourages cross-system improvements that positively impact speech and increases neural plasticity. The intensity and simple, focused cueing found in LSVT appears to be beneficial for patients with cognitive deficits resulting from congenital or acquired conditions.

The current case study examines the benefit of LSVT in a multilingual patient in their sixties with suspected progressive supranuclear palsy. At the time of the study, they exhibited a mild-moderate hypokinetic-hyperkinetic dysarthria characterized by reduced intelligibility, monoloudness, monopitch, and occasional rushes of accelerated speech. Vocal quality was hoarse, rough, and strained with occasional pitch breaks. In an oral motor evaluation, they demonstrated involuntary movements of their face, supporting the inclusion of the hyperkinetic component in the mixed dysarthria. The participant is a native French speaker who learned Spanish and English as second and third languages. Given the participant's known hypokinetic-hyperkinetic dysarthria, cognitive deficits, and multilingualism, we predicted that they would benefit from the intensity, structure, and simple cueing of LSVT. The goals of this case study were to determine the benefit of LSVT for improving speech production in English as a non-native language and for suspected progressive supranuclear palsy.

## Case description

The participant (pseudonym initials: OP) in this case study was a patient in their sixties at the time of treatment and was recruited from a university speech, language, and hearing sciences clinic. OP presented with atypical progressive supranuclear palsy per a neurologist's evaluation completed prior to participating in the study. Laryngoscopy examination indicated vocal fold bowing but no other laryngeal pathology. Speech-language evaluation conducted prior to treatment indicated mild-moderate aphasia (from testing using the Western Aphasia Battery-Revised in English, with informal administration of the picture description task in French also (15);, cognitive communication deficits (from testing using the Arizona Battery of Communication Disorders of Dementia (16);, and hypokinetic-hyperkinetic dysarthria (from perceptual speech evaluation). The cognitive-communication assessments were administered because OP was originally thought to have primary progressive aphasia, which was eventually ruled out. OP's dysarthria was characterized by reduced intelligibility, imprecise articulation, monoloudness, monopitch, reduced vocal quality (hoarse, rough, and strained), occasional rapid rate of speech with short rushes of speech, palilalia, and fatigue with longer periods of speech production. OP is multilingual in French, Spanish, and English, and they primarily use English for daily communication with occasional use of French at the time of the study. OP received individual speech-language therapy outside of the university clinic from 2019 to 2020, and both individual and group therapy at the university clinic beginning in the fall of 2020. Previous treatment targeted inspiratory and expiratory muscle strength training, use of attention and memory strategies, use of compensatory strategies to address word finding difficulties, improving sleep and exercise, engaging in daily activities that promote cognitive and language stimulation, independently asking follow-up questions during conversation, and independently expanding and elaborating on responses to questions during conversation.

## Diagnostic assessment

### Therapeutic intervention

LSVT is provided over the course of sixteen one-hour sessions conducted four days per week over the course of four consecutive weeks (9). Each one-hour session consists of daily activities, which include high intensity repetitions through a sustained vowel task, pitch glides up and down, production of a set of ten functional phrases, speech hierarchy tasks and spontaneous speech. The speech hierarchy tasks utilize materials and topics that are salient to the client, and gradually build from the word/phrase level in week one, to the sentence level in week two, to the paragraph level in week three, and finally the conversational level in week four. Calibration is addressed throughout each session and seeks to build the clients’ ability to self-cue use of adequate effort when producing loud speech, which in turn results in more intelligible speech. Homework consists of six repetitions each of the daily exercises (i.e., sustained phonation, pitch glides up, pitch glides down), repetition of functional phrases, and speech hierarchy practice. Homework is completed once per day on days the client receives treatment, and twice per day on days the client does not receive treatment. Adherence to the homework was assessed using a weekly homework tracking sheet and OP and their spouse would mark each week to help remind them to complete the program daily. This tracking sheet was reviewed each therapy session. In this case study, OP followed the typical LSVT protocol and completed sixteen LSVT treatment sessions over four weeks (Weeks 2–5 in Table 1). All sessions were completed in English with occasional words used in French (e.g., French cities, French foods, etc.).

**Table 1:** Treatment timeline.

Week	Activity
Week 1	Baseline measures taken
Week 2	Week 1 LSVT LOUD Administered
Week 3	Week 2 LSVT LOUD Administered
Week 4	Week 3 LSVT LOUD Administered
Week 5	Week 4 LSVT LOUD Administered; Immediate post data taken for acoustic measures and CES
Week 17	3-month post data taken for acoustic measures and CES

### Data collection

Speech was audio recorded during the baseline testing, post-treatment testing, and three-month follow-up to measure the effect of LSVT on acoustic features of speech for vocal quality (smooth cepstral peak prominence [CPPS] (17); and loudness control (mean intensity). CPPS was chosen as a measure of vocal quality in contrast to other potential measures (e.g., jitter, shimmer, harmonics-to-noise ratio, etc.) because it is the recommended measure by the American Speech Hearing Association as an index of dysphonia (18). All audio recordings were completed in English. Audio files were recorded in Audacity on a Dell laptop (XPS 15) with an AKG head-worn condenser microphone (C520) digitized through a MOTU UltraLite-mk3 Hybrid Audio Interface. The head-worn microphone was positioned one centimeter from the mouth. The audio files were then analyzed using Praat (19) to obtain data for intensity in dB SPL (mean, minimum, maximum) and smooth cepstral peak prominence. Acoustic measures were taken for the following tasks: sustained phonation, sentence repetition [from the CAPE-V sentences; (20)], reading of the Grandfather passage (21, 22), conversation, and picture description using the Cookie Theft image (23). Acoustic measures were analyzed for the entire audio sample for sustained phonation and sentence repetition, and for three randomly selected five second samples each from passage reading, conversation, and picture description.

To assess communication effectiveness, the Communicative Effectiveness Survey (CES) was completed by both the participant and their spouse at all phases of testing (pre-treatment, post-treatment, and three-month follow-up) (24).

### Perceptual analysis of intelligibility

Speech samples were extracted from assessments from pre-treatment, post-treatment, and three-month follow-up to conduct perceptual transcription of intelligibility. Three phrases were selected from four tasks: conversation, passage-reading, picture-description, and sentence repetition for each testing period. All trials were intensity normalized at 70 dB. Transcriptions of speech intelligibility were performed using Pavlovia from an experiment developed in PsychoPy (Version 2020.1.2) (25). The listeners included three speech-language pathologists with at least one year of experience in assessment of motor speech disorders. The listeners were instructed to transcribe the sample to the best of their ability. Intelligibility was then analyzed as the percentage of words transcribed correctly. The trials were randomly presented to the listeners.

### Statistical analysis

Statistical analyses were conducted with R version 4.0.5 (R Core Team, 2021) using RStudio version 1.4.1103 (RStudio Team, 2021). To analyze whether the treatment had an effect on acoustic measures of speech production, perceptual transcription of intelligibility, and responses to the CES, we ran two Bayesian general linear models using Stan modeling language (26) and the R package brms (27) (student family with an identify link estimated using MCMC sampling with 4 chains of 2000 iterations and a warmup of 1000). For these measures, the models separately predicted mean intensity, smooth cepstral peak prominence (CPPS), speech intelligibility, and patient rating scales for the CES by the interaction of treatment phase (baseline, post-treatment, and three-month maintenance) and speaking task (sustained phonation, sentence repetition, passage reading, picture description, and conversation) for the acoustic measures and speech intelligibility, and just treatment phase for CES responses. Non-informative priors were set for the models.

We report 95% credible intervals (CIs) and probability of direction for each effect. Probability of direction (pd) is the probability that a parameter is positive or negative (28). Given that a value of zero indicates no effect, a high pd value indicates a greater probability that the effect is greater than zero. The 95% CI means that we are 95% certain that the true value lies within a specified interval. A robust effect was considered one in which the 95% credible interval did not overlap with zero and that the PD was greater than 95%.

## Outcomes

### Acoustic measures

**Figure 1:**
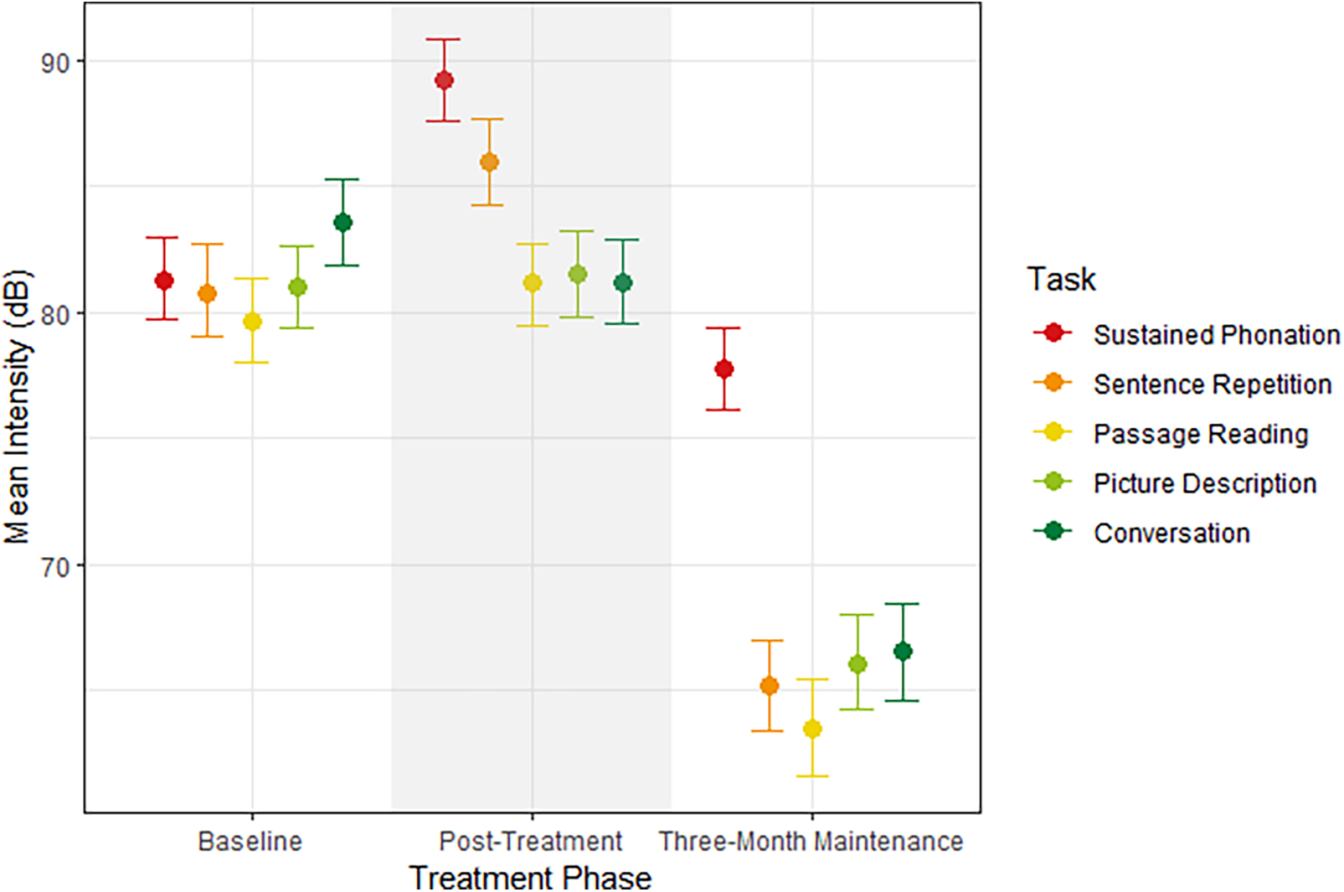
Median estimates and 95% credible intervals for mean intensity (dB) by treatment phase on the x-axis and speaking task.

The first model predicted mean intensity by treatment phase and speaking task. As shown in Figure 1, mean intensity increased from baseline to post-treatment by 2.53 dB [95% CI (1.47, 3.62); PD = 100%]. This effect was mainly driven by an increase in mean intensity for sustained phonation by 7.91 dB [95% CI (5.61, 10.23); PD = 100%] and sentence repetition by 5.20 dB [95% CI (2.78, 7.76); PD = 100%]. However, there was a robust decrease in intensity for conversation by 2.34 dB [95% CI (0.00, 4.81); PD = 97.02%]. There were no robust differences in passage reading or picture description although both increased in intensity. Overall, mean intensity increased after treatment but only robustly for the simple tasks of sustained phonation and sentence repetition and decreased for the more complex task of conversational speech.

During the three-month maintenance testing, mean intensity decreased from post-treatment testing by 15.98 dB [95% CI (14.77, 17.01); PD = 100%], indicating that the increased intensity during speech was not maintained. Mean intensity decreased across all tasks: sustained phonation by 11.41 dB [95% CI (9.10, 13.77); PD = 100%], sentence repetition by 20.78 dB [95% CI (18.56, 23.21); PD = 100%], passage reading by 17.63 dB [95% CI (15.16, 20.15); PD = 100%], picture description by 15.41 dB (95% CI [12.92, 18.00; PD = 100%), and conversation by 14.64 dB (95% CI [12.20, 17.19; PD = 100%).

**Figure 2:**
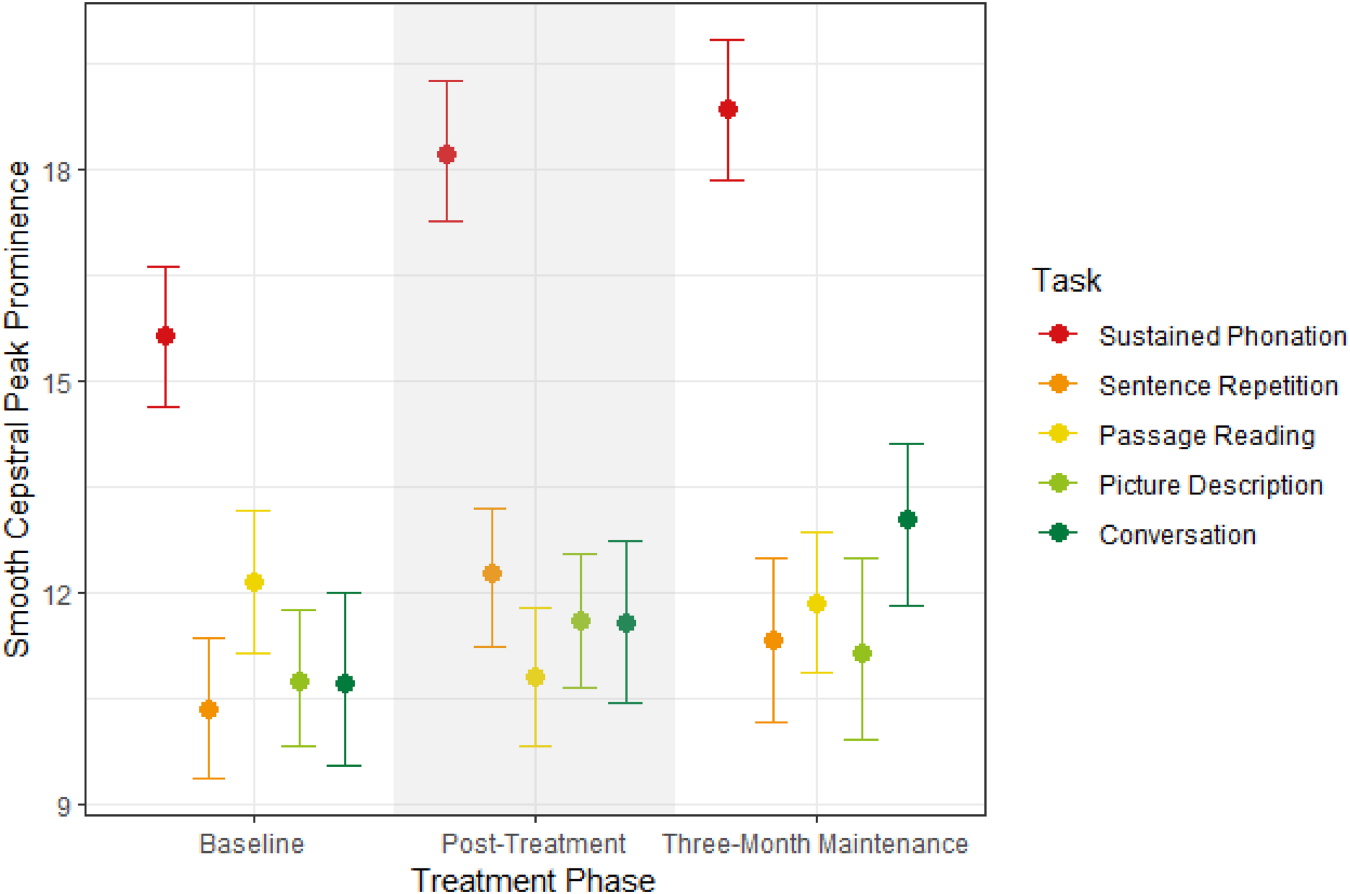
Median estimates and 95% credible intervals for CPPS by treatment phase on the x-axis and speaking task.

The second model predicted CPPS by treatment phase and speaking task. As shown in Figure 2, CPPS robustly increased from baseline to post-treatment testing by 0.98 dB [95% CI (0.29, 1.58); PD = 99.52%]. Specifically, sustained phonation increased from baseline to post-treatment by 2.60 dB [95% CI (1.26, 4.10); PD = 100%] as well as sentence repetition by 1.91 dB [95% CI (0.46, 3.35); PD = 99.08%]. Picture description increased but not robustly by 0.86 dB [95% CI (−0.58. 2.16); PD = 88.95%] as well as conversation by 0.85 dB [95% CI (−0.79, 2.45); PD = 85.40%]. Passage reading decreased by 1.34 dB [95% CI (−0.10, 1.70); PD = 97.08%]. Overall, treatment increased CPPS for all tasks except for passage reading.

Increases in CPPS were maintained during three-month testing, as evidenced by a non-robust overall increase by 0.34 dB [95% CI (−0.30, 1.04); PD = 84.38%]. Conversation increased robustly at three-month compared to post-treatment testing by 1.44 dB [95% CI (−0.28, 2.94); PD = 95.43%]. Increases in CPPS were maintained for sustained phonation at three-month testing by 0.63 dB [95% CI (−0.79, 1.96); PD = 83.3%] and passage reading by 1.01 dB [95% CI (−0.23, 2.50); PD = 93.67%]. CPPS decreased toward baseline levels for sentence repetition by 0.93 dB [95% CI (−0.85, 2.41); PD = 88.22%] and picture description by 0.46 dB [95% CI (−1.18, 2.05); PD = 71.38%], although not robustly. Overall, improvements in CPPS were maintained at three months, particularly for conversation, sustained phonation, and passage reading, but not for sentence repetition or picture description.

### Intelligibility

**Figure 3:**
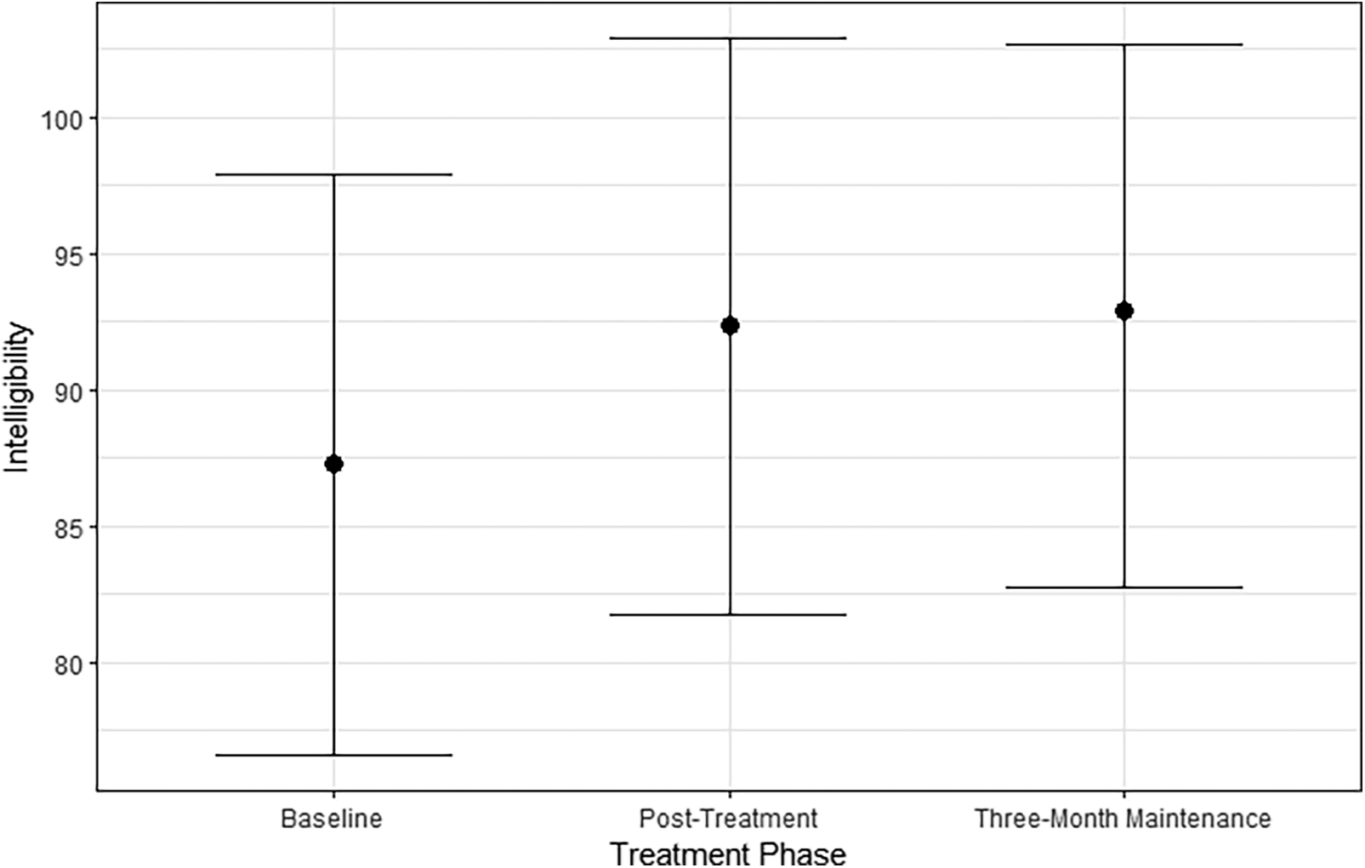
Median estimates and 95% credible intervals for intelligibility by treatment phase.

A Bayesian linear mixed model predicted perceived intelligibility by treatment phase and speaking task. As shown in Figure 3, the participant's intelligibility was relatively high at baseline at 87.21% [95% CI (76.04, 97.32)]. Intelligibility increased by 5.10% post-treatment [95% CI (−5.88, 16.18); PD = 81.58%]. The higher intelligibility was maintained in the three-month maintenance testing at 92.92% [95% CI (83.05, 100%)]. The difference from post-treatment to three-month testing was negligible [0.69%, 95% CI (−8.58, 10.10); PD = 55.53%]. Overall, the participant's intelligibility increased post-treatment, but the effect was small because their intelligibility was already high at baseline. There were no robust effects of speaking task by treatment phase.

### Communicative effectiveness

A Bayesian generalized linear model predicted CES responses by treatment phase and respondent. CES responses increased from baseline to post-treatment testing for both the participant and the spouse by 0.71 points [95% CI (0.17, 1.24); PD = 99.25%], indicating that both respondents found the treatment to be beneficial for communicative effectiveness. At three-month maintenance testing, responses on the CES decreased overall by 0.39 points [95% CI (−0.14, 0.94); PD = 92.40%]. Overall, improvements in communicative effectiveness were not maintained. There were no robust interactions by phase and respondent, indicating that both the participant and spouse had similar ratings at each phase. The full table of the CES responses by item are listed in Supplementary Table S1.

## Discussion

This study sought to determine whether LSVT LOUD would improve vocal quality, speech intelligibility, and communicative effectiveness for a multilingual patient seeking treatment for hypokinetic-hyperkinetic dysarthria secondary to suspected progressive supranuclear palsy. Immediately post-treatment, vocal intensity and vocal quality improved for less complex speaking tasks (e.g., sustained phonation and sentence repetition), as well as overall intelligibility. Additionally, the participant and their spouse's perception of communication effectiveness improved post-treatment. Three months post treatment, positive changes in vocal quality and intelligibility were maintained. However, positive changes in vocal intensity were not maintained.

The main acoustic findings of this study were that vocal quality robustly increased post-treatment during sustained phonation and sentence repetition, as well as less robustly for picture description and conversation tasks. Three months post treatment, gains in vocal quality were maintained across these tasks and improved beyond immediate post-treatment levels in conversation. Vocal intensity increased for sustained phonation and sentence repetition immediately post-treatment. Three months post treatment, vocal intensity dropped below baseline levels on all tasks. Overall, LSVT improved vocal quality for OP and they maintained that improvement. Improvements in vocal intensity were observed post-treatment but not maintained.

Immediately post-treatment, vocal quality and vocal intensity improved in simpler, less cognitive-demanding speaking tasks. The higher cognitive load required in more complex speaking tasks (e.g., conversation, reading, picture description), as well as the effects of fatigue in tasks with longer duration, are possible explanations for less consistent use of target voice during more complex speaking tasks. OP demonstrated increased self-cueing of target voice during treatment sessions per clinician observation, particularly in less complex speaking tasks. It is also possible that due to the higher cognitive load and duration of the activity, that OP performed self-cueing behaviors less frequently in more complex tasks. It is also important to consider how difficult the reading tasks may have been for OP. Untreated diplopia, paired with a possible history of dyslexia, English as their third language and cognitive deficits made reading a particularly difficult task for them.

The improvement in vocal quality observed three-months post treatment suggests increased generalization and automaticity of target voice during conversational speech. OP's voice had a hoarse, rough, and strained vocal quality prior to treatment, and treatment sessions focused on shaping a level of loudness that supported a healthy vocal quality. Given the shaping used in sessions, the time since intensive treatment, and OP reports of no longer completing daily homework tasks, it is unsurprising that a decrease in vocal intensity was found.

Intelligibility increased post-treatment and was maintained at three-month testing. At baseline, intelligibility was relatively high at 87%, so although increases in intelligibility were not robust and included quite a bit of uncertainty, the overall increase to 91% could be clinically meaningful if the increase assists with being understood by listeners. There was no interaction of task by intelligibility, meaning that the increase in intelligibility occurred across tasks. Overall, LSVT was a beneficial treatment for improving intelligibility for our case study but not overall speech severity. This result could be because understandability of speech improved more than prosodic production.

Improved communicative effectiveness was indicated by OP's and their spouse's responses to the CES immediately post-treatment. The overall score improved in both participant and spouse reports, indicating increased communicative effectiveness. OP reported gains in conversing with family and friends at home, conversing with a stranger on telephone, conversing in noisy gatherings, and talking in the car following LSVT LOUD treatment. However, these gains were not maintained when the CES was readministered three months post-treatment. Both OP and spouse noted a decrease in communicative effectiveness three months following treatment. OP's score returned to pretreatment levels, and the spouse's score to one point above pretreatment levels. A multitude of factors, such as no longer maintaining a home practice routine, changes in the family's personal circumstances, shifts in available social activities due to seasonal changes, and changes in expectations of performance may have played a role in the change in scores. It is also possible that the treatment gave a temporary inflated boost of communication confidence.

While the results of this study are promising overall for the benefit of LSVT for this patient, one limitation of this study was that the training and assessments were not completed in the patient's first language (French). However, English has been the patient's dominant language since moving to the United States over thirty years prior, and English is the language they use to communicate for most of their needs throughout the day. Therefore, for functionality, English was the best choice to use for this therapy, given that they do not use French for daily tasks. Furthermore, some French was used in training in the form of French words, French cities, French wines, and French phrases. Therefore, even though a majority of the training was completed in English, some of the training was completed in French and used functional words and phrases in both languages. Choosing which language to use in therapy is an important decision for clinicians: it is important to consider both fluency and everyday use of each language, regardless of which language was spoken first by the patient.

A likely explanation for the lack of maintenance of the treatment effects is the progression of the patient's disease. If the diagnosis of progressive supranuclear palsy is correct, then progression is rapid, with a survival rate of 6–7 years post-onset (29). With a rapidly progressing disease, it is likely that speech therapy may be able to temporarily improve speech, maintain the current level of speech, or slow down the progression of speaking impairments. However, it is likely that any improvements from speech therapy will be diminished as the disease progresses. Regardless, it is still important to pursue speech therapy in these progressive diseases so that communication and social participation can be maintained.

These results appear to be in line with results in other LSVT studies. In available studies with maintenance data, a decline in communicative effectiveness in the maintenance period is common (30, 31). Additionally, many participants in these studies reported a lack of compliance with the home practice program six months post treatment in the study. Reasons for lack of compliance are not provided but potential reasons could include the time commitment of the home practice program and the lack of accountability following treatment.

## Limitations

The findings from this study, while informative, have limitations in generalizability. First, this is a case study with one patient, so the findings need to be replicated with additional multilingual patients, and other patients with progressive supranuclear palsy. Second, patient adherence to homework was assessed from patient report. It is possible that actual adherence was different from what was reported based on potential reporting bias in wanting to report positive rather than negative adherence. Third, it would have been helpful to have more holistic information about the patient, including the patient's main concerns and past medical, family, or psychosocial history. However, that information was not obtained and, therefore, could not be included in this case study. Furthermore, it would have been beneficial to have the patient's perspective on why communicative effectiveness declined three months post-treatment; however, this information was not obtained at that time. Fourth, it would have been beneficial to compared acoustic measures in English and French to see how well the therapy generalized to his speech in French. Future studies of multilingual clients should consider obtaining speech samples across languages to determine generalizability.

## Conclusion

LSVT was beneficial in improving vocal quality, vocal loudness, intelligibility, and communicative effectiveness in a multilingual man with hypokinetic-hyperkinetic dysarthria secondary to suspected progressive supranuclear palsy. Improvements in vocal quality, intelligibility, and quality of life were maintained three months post-treatment. Clinicians who work with complex patients may wish to consider the theoretical underpinnings of LSVT LOUD, client profile, and areas of client need, and ability and desire to complete a 16-week treatment program to determine if trialing LSVT LOUD is appropriate. The use of LSVT LOUD with complex clients may yield positive outcomes.

## Data Availability

The datasets presented in this study can be found in online repositories. The names of the repository/repositories and accession number(s) can be found below: https://osf.io/cx8na/.

## References

[1] Ramig LO Fox C Sapir S. Parkinson’s disease: speech and voice disorders and their treatment with the Lee Silverman voice treatment. Semin Speech Lang. (2004) 25(2):169–80. 10.1055/s-2004-825653 15118943

[2] Blumin JH Pcolinsky DE Atkins JP. Laryngeal findings in advanced Parkinson’s disease. Annals of otology. Rhinology & Laryngology. (2004) 113(4):253–8. 10.1177/000348940411300401 15112966

[3] Skodda S Visser W Schlegel U. Vowel articulation in Parkinson’s disease. J Voice. (2011) 25(4):467–72. 10.1016/j.jvoice.2010.01.009 20434876

[4] D’Arrigo A Floro S Bartesaghi F Casellato C Papa GFS Centanni S Respiratory dysfunction in Parkinson’s disease: a narrative review. ERJ Open Res. (2020) 6:4. 10.1183/23120541.00165-2020 PMC7533305 33043046

[5] Baumann A Nebel A Granert O Giehl K Wolff S Schmidt W Neural correlates of hypokinetic dysarthria and mechanisms of effective voice treatment in Parkinson disease. Neurorehabil Neural Repair. (2018) 32(12):1055–66. 10.1177/1545968318812726 30444176

[6] Mahler LA Jones HN. Intensive treatment of dysarthria in two adults with down syndrome. Dev Neurorehabil. (2012) 15(1):44–53. 10.3109/17518423.2011.632784 22256834

[7] Mahler LA Ramig LO. Intensive treatment of dysarthria secondary to stroke. Clin Linguist Phon. (2012) 26(8):681–94. 10.3109/02699206.2012.696173 22774928

[8] Moya-Galé G Goudarzi A Bayés À McAuliffe M Bulté B Levy ES. The effects of intensive speech treatment on conversational intelligibility in spanish speakers with Parkinson’s disease. Am J Speech Lang Pathol. (2018) 27(1):154–65. 10.1044/2017_AJSLP-17-0032 29351354

[9] Sapir S Ramig LO Fox CM. Intensive voice treatment in Parkinson’s disease: Lee Silverman voice treatment. Expert Rev Neurother. (2011) 11(6):815–30. 10.1586/ern.11.43 21651330

[10] Whitehill TL Kwan L Lee FP-H Chow MM-N. Effect of LSVT on lexical tone in speakers with Parkinson’s disease. Parkinson’s Disease. (2011) 2011:e897494. 10.4061/2011/897494 21876841 PMC3163132

[11] Crystal D. English as a Global Language. Cambridge, UK: Cambridge University Press (2003).

[12] Baldanzi C Crispiatico V Foresti S Groppo E Rovaris M Cattaneo D Effects of intensive voice treatment (the Lee Silverman voice treatment [LSVT LOUD]) in subjects with multiple sclerosis: a pilot study. J Voice. (2022) 36(4):585.e1–585.e13. 10.1016/j.jvoice.2020.07.025 32819780

[13] Sapir S Pawlas A Ramig L Seeley E Fox C Corboy J. Effects of intensive phonatory-respiratory treatment (LSVT) on voice in individuals with multiple sclerosis. NCVS Stat Prog Rep. (1999) 14:141–7.

[14] Sale P Castiglioni D De Pandis MF Torti M Dall’Armi V Radicati FG The Lee Silverman voice treatment (LSVT®) speech therapy in progressive supranuclear palsy. Eur J Phys Rehabil Med. (2015) 51:569–74. . 26138088

[15] Kertesz A. Western aphasia battery—Revised (2007).

[16] Bayles KA Tomoeda CK. Arizona Battery for Communication Disorders of Dementia. Bellemont, AZ: Canyonlands Publishing (1993). 10.3109/13682829609042219 8776438

[17] Murton O Hillman R Mehta D. Cepstral peak prominence values for clinical voice evaluation. Am J Speech Lang Pathol. (2020) 29(3):1596–607. 10.1044/2020_AJSLP-20-00001 32658592 PMC7893528

[18] Patel RR Awan SN Barkmeier-Kraemer J Courey M Deliyski D Eadie T Recommended protocols for instrumental assessment of voice: american speech-language-hearing association expert panel to develop a protocol for instrumental assessment of vocal function. Am J Speech Lang Pathol. (2018) 27(3):887–905. 10.1044/2018_AJSLP-17-0009 29955816

[19] Boersma P Weenink D. Praat: Doing phonetics by computer [Computer program], Version 6.0.46. (2019). Available online at: http://www.Praat.Org

[20] Solomon NP Helou LB Stojadinovic A. Clinical versus laboratory ratings of voice using the CAPE-V. J Voice. (2011) 25(1):e7–e14. 10.1016/j.jvoice.2009.10.007 20430573

[21] Reilly J Fisher JL. Sherlock holmes and the strange case of the missing attribution: a historical note on “the grandfather passage.”. J Speech Lang Hear Res. (2012) 55(1):84–8. 10.1044/1092-4388(2011/11-0158) 22354714

[22] Van Riper C. Speech Correction; Principles and Methods. Hoboken, NJ: Prentice-Hall (1963).

[23] Goodglass H Kaplan E Barresi B. BDAE3 Boston Diagnostic Aphasia ExaminationThird Edition. (2001). Available online at: https://www.proedinc.com/Products/11850/bdae3-boston-diagnostic-aphasia-examinationthird-edition.aspx (Accessed January 7, 2021).

[24] Donovan NJ Velozo CA Rosenbek JC. The communicative effectiveness survey: investigating its item-level psychometric properties. Am J Speech Lang Pathol. (2007) 15(4):433–48.

[25] Peirce J. PsychoPy-Psychology software for Python Release 2020.1.2 (2020).

[26] Carpenter B Gelman A Hoffman MD Lee D Goodrich B Betancourt M Stan: a probabilistic programming language. J Stat Softw. (2017) 76:1. 10.18637/jss.v076.i01 36568334 PMC9788645

[27] Bürkner P-C. Brms: an R package for Bayesian multilevel models using stan. J Stat Softw. (2017) 80(1):1–28. 10.18637/jss.v080.i01

[28] Makowski D Ben-Shachar MS Chen SHA Lüdecke D. Indices of effect existence and significance in the Bayesian framework. Front Psychol. (2019) 10:2767. 10.3389/FPSYG.2019.02767 31920819 PMC6914840

[29] Arena JE Weigand SD Whitwell JL Hassan A Eggers SD Höglinger GU Progressive supranuclear palsy: progression and survival. J Neurol. (2016) 263(2):380–9. 10.1007/s00415-015-7990-2 26705121

[30] Ramig L Halpern A Spielman J Fox C Freeman K. Speech treatment in Parkinson’s disease: randomized controlled trial (RCT). Mov Disord. (2018) 33(11):1777–91. 10.1002/mds.27460 30264896 PMC6261685

[31] Wenke RJ Theodoros D Cornwell P. The short- and long-term effectiveness of the LSVT®for dysarthria following TBI and stroke. Brain Inj. (2008) 22(4):339–52. 10.1080/02699050801960987 18365848

