# Supplementary material for: Case Report: Benefits of LSVT LOUD in a multilingual patient with hypokinetic-hyperkinetic dysarthria and suspected progressive supranuclear palsy
Source: Front Rehabil Sci. 2024 Jul 18;5:1421730. doi: 10.3389/fresc.2024.1421730 (PMC11291323; doi:10.3389/fresc.2024.1421730)
Supplement: Supplementary file 1 [file Table1.docx]

**Supplementary Table 1.** Item responses for the Communicative Effectiveness Survey for the participant and the spouse by treatment phase.

| **Communicative Effectiveness Survey (CES)** | | | | | | |
| --- | --- | --- | --- | --- | --- | --- |
| Key: 1= not at all effective, 4= very effective | | | | | | |
|  | **Baseline** | | **Immediately Post-treatment** | | **3 Months Post** | |
| Item | Participant Response | Spouse Response | Participant Response | Spouse Response | Participant Response | Spouse Response |
| 1. Having a conversation with a family member or friends at home. | 2 | 3 | 3 | 3 | 3 | 3 |
| 1. Participating in conversation with strangers in a quiet place. | 2 | 2 | 2 | 3 | 2 | 2 |
| 1. Conversing with a familiar person over the telephone. | 3 | 3 | 3 | 3 | 3 | 3 |
| 1. Conversing with a stranger over the telephone. | 1 | 1 | 3 | 2 | 2 | 2 |
| 1. Being part of a conversation in a noisy environment (social gathering). | 1 | 2 | 3 | 2 | 2 | 1 |
| 1. Speaking to a friend when you are emotionally upset or angry. | 4 | 2 | 3 | 3 | 3 | 2 |
| 1. Having a conversation while travelling in a car. | 2 | 3 | 3 | 4 | 4 | 3 |
| 1. Having a conversation with someone at a distance (across a room). | 2 | 1 | 3 | 2 | 2 | 2 |
| **Total** | **17** | **17** | **23** | **22** | **17** | **18** |
